# The Added Value of 4D‐Flow CMR: Identifying Aortic Regurgitation as the Culprit for Severe Cardiomyopathy

**DOI:** 10.1002/ccr3.72074

**Published:** 2026-02-20

**Authors:** Alexander Gall, Pankaj Garg

**Affiliations:** ^1^ Department of Cardiovascular and Metabolic Health, Norwich Medical School University of East Anglia Norfolk UK; ^2^ Department of Cardiology Norfolk and Norwich University Hospitals NHS Foundation Trust Norwich UK

**Keywords:** 4D‐flow, aortic regurgitation, cardiac magnetic resonance, cardiomyopathy

## Abstract

Hemodynamically significant aortic regurgitation (AR) may be underestimated by standard echocardiography and 2D phase‐contrast CMR, particularly in patients with heart rate variability. This case highlights the additive role of 4D‐flow CMR in reclassifying AR severity, thereby identifying a significant pathology and helping to guide timely intervention.

## 1 Case Description

A 73‐year‐old female was admitted to the hospital with progressive shortness of breath. Her medical history was notable for breast cancer, treated successfully 19 years prior. She was diagnosed with acute decompensated heart failure and treated acutely with intravenous furosemide.

Initial investigations revealed a significantly elevated NT‐proBNP of 5772 ng/L (normal range < 400). The electrocardiogram showed sinus rhythm with a narrow QRS complex. A transthoracic echocardiogram (TTE) demonstrated a dilated left ventricle (LV) with severe global systolic dysfunction (estimated ejection fraction [EF] 10%–20%). The TTE also identified moderate aortic regurgitation (AR), though the aortic valve appeared morphologically normal with normal aortic dimensions. Prior to discharge, she was initiated on guideline‐directed medical therapy (GDMT) including bisoprolol, ramipril, spironolactone, and dapagliflozin.

To further investigate the etiology of her nonischemic cardiomyopathy, an outpatient cardiac magnetic resonance (CMR) study was performed 2 months after her index admission. The CMR confirmed severe LV dilation (end‐diastolic volume index 159 mL/m^2^) and severe systolic dysfunction (LVEF 26%). The right ventricle (RV) was normal in size but had impaired systolic function (RVEF 41%).

**FIGURE 1:**
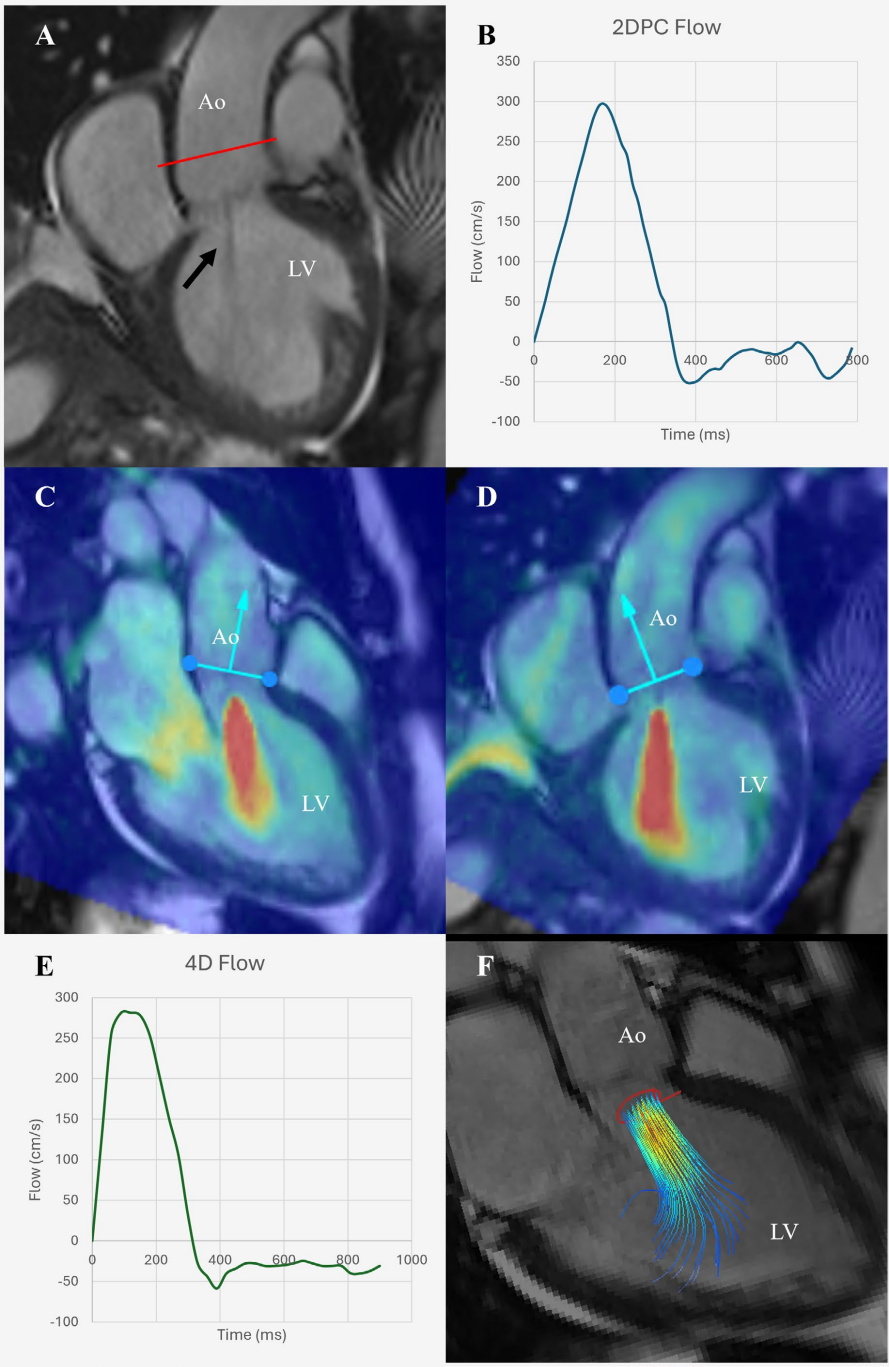
Comprehensive cardiac magnetic resonance (CMR) assessment of aortic regurgitation. Panel A: Cine image (LVOT view) in mid‐diastole. Red line indicates planning for a 2D phase contrast sequence. Central jet of aortic regurgitation visible (arrow). Panel B: Flow curve analysis from 2D phase‐contrast sequence (RF = 20%). Panels C and D: 4D‐flow velocity mapping overlaid onto three‐chamber cine (C) and LVOT cine (D), demonstrating a central jet of severe aortic regurgitation. Panel E: 4D‐flow flow‐curve analysis of AR jet, demonstrating holodiastolic flow reversal and reclassifying AR severity. RF = 38%. Panel F: three‐dimensional volume‐rendered 4D‐flow analysis demonstrating a central regurgitant jet. AR, aortic regurgitation; LVOT, left ventricular outflow tract; RF, regurgitant fraction.

AR severity analysis with standard 2D phase‐contrast quantification indicated mild‐to‐moderate AR (regurgitant fraction [RF] 20%, regurgitant volume [RVol] 10.9 mL) (Figure 1A,B). In contrast, advanced 4D‐flow analysis provided a critical diagnostic revision, demonstrating moderate‐to‐severe AR (RF 38%, RVol 20 mL) (Figure 1C–1F). The etiology of the AR is not definitively explained from the available imaging.

Furthermore, tissue characterization provided evidence of established adverse remodeling. There was diffuse myocardial fibrosis (elevated native T1 (1083 ms [normal 920–1050]) and extracellular volume [32%]) and subtle mid‐wall late gadolinium enhancement (LGE).

The patient remains under workup for their cardiomyopathy, including referral to the inherited cardiac conditions service, prior to consideration of intervention for their AR.

## 2 Discussion

This case demonstrates the significant additive diagnostic value of a comprehensive CMR protocol incorporating 4D‐flow. The 4D‐flow analysis reclassified the patient's AR from mild‐moderate to moderate‐severe, fundamentally changing the clinical picture, while also providing the well described additive benefit to TTE of tissue characterization [1]. This finding raises the question of whether the AR is the primary driver of this patient's cardiomyopathy, or a secondary consequence, while also indicating the need for more regular surveillance and possible intervention. Underestimation with 2D phase‐contrast may be due to significant heart rate variability as was seen in this patient, with 4D‐flow averaging over a longer time period therefore improving accuracy and reliability.

Moreover, the presence of mid‐wall LGE and elevated T1/ECV mapping confirms established, adverse LV remodeling. These fibrotic changes may represent the chronic sequelae of volume overload that timely aortic valve intervention aims to prevent [2].

This case highlights the diagnostic challenge with hemodynamically significant AR and the additive value of advanced imaging as part of a multimodality assessment. Current ESC guidelines support referral for intervention in view of the negative remodeling in this case, while a recent meta‐analysis has suggested a lower ARF of > 33% on CMR as a threshold for intervention, irrespective of negative remodeling [1, 2, 3].

## Author Contributions

**Alexander Gall:** conceptualization, data curation, formal analysis, methodology, writing – original draft, writing – review and editing. **Pankaj Garg:** conceptualization, supervision, writing – review and editing.

## Funding

The authors have nothing to report.

## Consent

The authors confirm that consent has been obtained for publication of this case.

## Conflicts of Interest

The authors declare no conflicts of interest.

## Data Availability

Data sharing is not applicable to this article because no datasets were generated or analysed during the preparation of this case.
